# Inferring the Disease-Associated miRNAs Based on Network Representation Learning and Convolutional Neural Networks

**DOI:** 10.3390/ijms20153648

**Published:** 2019-07-25

**Authors:** Ping Xuan, Hao Sun, Xiao Wang, Tiangang Zhang, Shuxiang Pan

**Affiliations:** 1 School of Computer Science and Technology, Heilongjiang University, Harbin 150080, China; 2 School of Computer Science, Beijing University of Posts and Telecommunications, Beijing 100876, China; 3 School of Mathematical Science, Heilongjiang University, Harbin 150080, China

**Keywords:** disease-associated miRNAs, network representation learning, convolutional neural network, non-negative matrix factorization, deep learning

## Abstract

Identification of disease-associated miRNAs (disease miRNAs) are critical for understanding etiology and pathogenesis. Most previous methods focus on integrating similarities and associating information contained in heterogeneous miRNA-disease networks. However, these methods establish only shallow prediction models that fail to capture complex relationships among miRNA similarities, disease similarities, and miRNA-disease associations. We propose a prediction method on the basis of network representation learning and convolutional neural networks to predict disease miRNAs, called CNNMDA. CNNMDA deeply integrates the similarity information of miRNAs and diseases, miRNA-disease associations, and representations of miRNAs and diseases in low-dimensional feature space. The new framework based on deep learning was built to learn the original and global representation of a miRNA-disease pair. First, diverse biological premises about miRNAs and diseases were combined to construct the embedding layer in the left part of the framework, from a biological perspective. Second, the various connection edges in the miRNA-disease network, such as similarity and association connections, were dependent on each other. Therefore, it was necessary to learn the low-dimensional representations of the miRNA and disease nodes based on the entire network. The right part of the framework learnt the low-dimensional representation of each miRNA and disease node based on non-negative matrix factorization, and these representations were used to establish the corresponding embedding layer. Finally, the left and right embedding layers went through convolutional modules to deeply learn the complex and non-linear relationships among the similarities and associations between miRNAs and diseases. Experimental results based on cross validation indicated that CNNMDA yields superior performance compared to several state-of-the-art methods. Furthermore, case studies on lung, breast, and pancreatic neoplasms demonstrated the powerful ability of CNNMDA to discover potential disease miRNAs.

## 1. Introduction

MicroRNAs (miRNAs) are a class of endogenous small RNAs of approximately 20–24 nucleotides in length. miRNAs regulate gene expression in plants and animals after transcription [1,2,3]. Accumulating studies indicate that miRNAs are closely related to the development of human diseases [4,5,6,7]. Therefore, it is imperative to explore potential disease-associated miRNAs (disease miRNAs) in order to understand disease etiology and pathogenesis.

Disease miRNAs prediction can provide reliable candidates for experimental research. Several methods have been proposed for predicting potential disease miRNAs. Mainstream methods are roughly grouped into two categories. The first category of methods primarily uses the regulatory relationship between miRNAs and their target mRNA to predict potential miRNA-disease associations [8]. First, target genes related to miRNAs are obtained by analyzing base complementarity between the miRNA sequence and the putative target gene sequence. Then, using the interactions between the target gene and known disease-related genes, the potential disease miRNAs are predicted [9,10,11,12]. However, such methods are difficult to use due to experimentally validated targets being insufficiently described to date. Although more target gene samples were obtained through some experiments [13,14], prediction results from these methods have a high false positive rate.

Methods belonging to the second category are based on prior biological knowledge that miRNAs with similar functions are usually associated with similar diseases [15]. First, network medicine is the mainstream way of defining related diseases [16,17,18], some methods make full use of network topology to identify disease miRNAs [19,20]. Moreover, disease miRNAs are identified by a random walk on a single miRNA similarity network [21,22]. However, these methods rely too much on known disease-associated miRNAs and are ineffective for new diseases that lack associated miRNAs. To address this drawback, disease similarity information and miRNA-disease associations were introduced to form miRNA-disease heterogeneous networks, where random walks on a two-layer network were used to predict candidate miRNA-disease associations [23,24]. In addition, there are other methods available for calculating miRNA-disease correlation scores, several methods use non-negative matrix factorization [25,26,27,28,29]. By applying structural perturbation [30], by using transduction learning [31], by using the induction matrix [32], through the binary network projection [33], and extracting potential features that pertain to positive sample information [34]. However, there are complex and non-linear relationships between miRNA-miRNA, disease-disease, and miRNA-disease, all previous methods struggle to extract such relationships.

In this study, we present a new approach on the basis of convolutional neural networks for predicting miRNA-disease association, called CNNMDA. It contains two parts consisting of a left and a right. CNNMDA’s left part deeply integrates miRNA similarities, disease similarities, and miRNA-disease associations, and uses these prior biological knowledge to construct the left embedding layer of the miRNA-disease node pair. The right part uses network representation learning to obtain a potential low-dimensional representation of the network node while preserving the topology of the network. Integrating the low dimensional features of miRNAs and diseases helps to estimate the likelihood of association between miRNAs and diseases at the global network level. We construct a deep learning framework based on convolutional neural networks (CNN) for the left and right parts, and learn the original representation and global representation of miRNA-disease node pairs. For some high-frequency diseases, CNNMDA can determine them with high accuracy. Moreover, case studies on 3 diseases indicate that CNNMDA is able to discover potential disease associated miRNAs.

## 2. Results and Discussion

### 2.1. Evaluation Metrics

To evaluate the performance of our prediction model, we performed a 5-fold cross-validation on CNNMDA. In the miRNA-disease association data set, the known miRNA-disease associations are called positive samples, while the unknown associations are considered negative samples. In the first place, all positive samples were extracted, and were divided into five subsets randomly. The next step was to extract the same number of negative samples as the positive samples, and these negative samples are also divided into five subsets randomly. In each cross-validation, we took four positive and four negative samples from five subsets to train the prediction model, and the remaining one positive sample and one negative sample were used as test data to evaluate the prediction performance.

Given a threshold τ, a positive sample is obtained when the prediction score is higher than τ, otherwise a negative sample is added. Accordingly, TPR and FPR are calculated by the following formula:

(1) $$\mathrm{TPR}=\frac{\mathrm{TP}}{\mathrm{TP}+\mathrm{FN}},\;\mathrm{FPR}=\frac{\mathrm{FP}}{\mathrm{TN}+\mathrm{FP}},$$

where TP and TN represent the number of positive and negative samples that are judged correctly, respectively. FN indicates the number of positive samples that are misidentified as negative samples, and FP represents the number of negative samples that are misidentified as positive examples. We can calculate different TPRs and FPRs based on different thresholds. The obtained TPRs and FPRs can be plotted as ROC curves, and the area under the receiver operating characteristic curve (AUC) can be used as a criterion for evaluating prediction performance.

By observing relevant data, we noted that there were only a few known miRNA-disease associations (positive samples), accounting for 131 of all associated data. It is not difficult to surmise that there is a serious imbalance between positive samples and negative samples. In this case, the PR (precision-recall) curve usually reflects more information than the ROC curve [35,36]. Precision indicates the proportion of positive samples that are defined correctly compared to the number of positive samples currently defined as positive examples. Recall indicates the proportion of positive samples that are defined correctly compared to all positive samples. This is calculated as follows:

(2) $$\mathrm{Precision}=\frac{\mathrm{TP}}{\mathrm{TP}+\mathrm{FP}},\;\mathrm{Recall}=\frac{\mathrm{TP}}{\mathrm{TP}+\mathrm{FN}}.$$

Similarly, precisions and recalls are calculated by different thresholds. Based on these values, the PR curve can be plotted and the area under the precision–recall curve (AUPR) can be calculated to evaluate the prediction performance of the model. In addition, biologists usually choose the top-rank prediction results for experimental validation, so we calculated the average recall value for 15 diseases in the top k∈{30, 60, 90,…, 240} as another evaluation method.

### 2.2. Comparison with Other Method

To evaluate the prediction performance of CNNMDA, we compare it with several methods that are at the forefront of the field. These included DMPred [29], GSTRW [37], BNPMDA [33], and Liu’s method [23], where the parameter settings for each method were set to achieve the best performance. In CNNMDA, the parameters $w_{l}$, $w_{f}$, and $w_{p}$ in the convolution operation were set to 3, 5, and 2, respectively. Thus, the size of the convolution sliding window $J\in R^{3\times 5}$, and the sliding window $F\in R^{1\times 2}$ in the pooling operation. The number of filters was set to 30 (n_conv_ = 30). The parameters $\alpha ,\;\beta ,{\;\lambda }_{m},{\;\mathrm{and}\;\lambda }_{d}$. used in the matrix factorization were all obtained from the set {0.2, 0.5, 0.8, 1, 2, 5, 8} by cross-validating the values of the various parameters. CNNMDA achieved the best performance when α=0.2, β=0.2, $\lambda_{m}=0.2$, and $\lambda_{d}=0.2$. In addition, the parameter λ in the combination formula for the left part and right part was set to 0.4. In other comparison methods, the parameters are set according to the values given in the original article.

**Figure 1:**
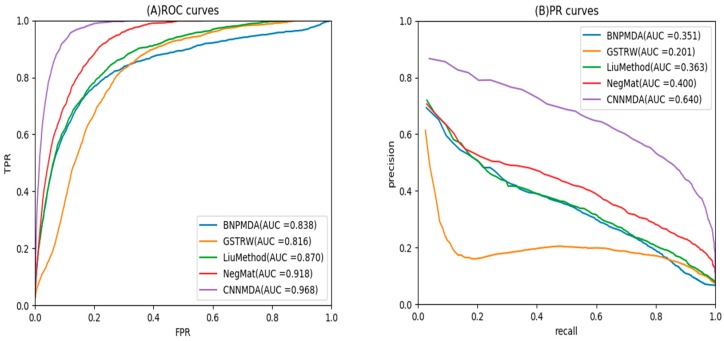
ROC curves and precision-recall (PR) curves of CNNMDA and other methods for 15 diseases.

As shown in Figure 1A and Table 1, CNNMDA achieved the best average performance for 15 diseases (AUC of ROC curve = 0.968). DMPred’s performance was the second best, where the AUC was 5% lower than CNNMDA, reaching 0.918. In addition, the AUC values of BNPMDA and Liu reached 0.838 and 0.870, which were 13% and 9.8% lower than CNNMDA, respectively. GSTRW performed poorly compared with other methods, and its AUC value was only 0.816, 15.2% lower than CNNMDA. Among the methods, GSTRW displayed poor performance since only miRNA and disease similarity information is used in this method. Liu’s method and BNPMDA fully capture the information of the network topology, and DMPred improves performance by integrating multiple sources of effective information. Our method, CNNMDA, through deep learning original representation and global representation of miRNA-disease node pairs, achieved the best prediction performance. CNNMDA also obtained the best results in each disease.

As shown in Figure 1B and Table 2, we obtained the average AUPR of all the methods with respect to 15 diseases, and plotted the corresponding PR curves. It is not difficult to surmise that the average AUC-PR area of CNNMDA under 15 diseases was also significantly higher than for other methods. Compared with GSTRW, BNPMDA, Liu’s Method and DMPred, CNNMDA displayed AUC-PR increases of 43.9%, 28.9%, 27.7%, and 24%, respectively. Moreover, in 13 of the 15 diseases, CNNMDA achieved the best performance.

In addition, to further verify the superior performance of our method compared with other methods, we applied a commonly used method called a paired *t*-test. After calculation, the *p*-values of all paired *t*-test results were less than 0.05 (Table 3), indicating that the performance of CNNMDA is significantly better than other methods.

**Figure 2:**
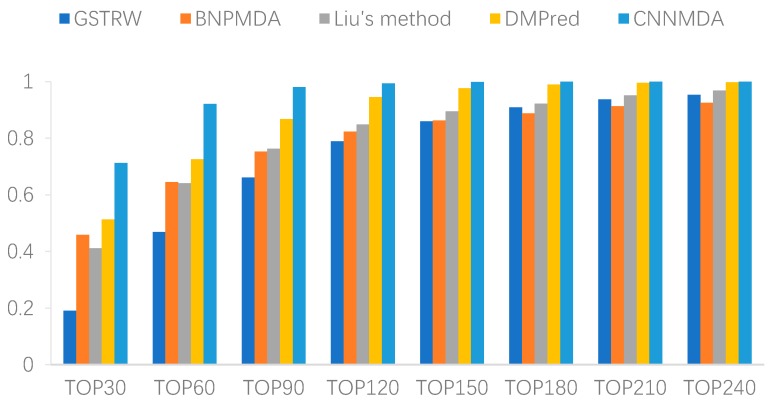
Recall values of top *k* candidates of CNNMDA and the other four methods.

This was accompanied by a higher recall rate, which means that we have successfully identified more positive samples in the top *k* candidate list, further indication of the superiority of this model’s prediction performance. Therefore, we calculated the average recall rate for all methods in 15 diseases (Figure 2). Our method achieved the highest average recall rate at different thresholds, where the top 30 reached 0.712, the top 60 reached 0.921, and the top 90 reached 0.980. The recall rate of DMPred was the second best at all thresholds, and ranked 0.512 in the top 30, 0.726 in the top 60, and 0.860 in top 90. The recall rate of BNPMDA and Liu was very close. The average recall rates of the top 30, the top 60, the top 90 in the former were 0.459, 0.645, and 0.753, and the latter were 0.411, 0.641, and 0.763, respectively. In contrast, GSTRW exhibited poor performance, and the recall rates in the top 30, top 60 and top 90 were 0.191, 0.469, and 0.661, respectively.

### 2.3. Case Studies of Lung Neoplasms, Breast Neoplasms, and Pancreatic Neoplasms

To demonstrate CNNMDA’s ability to discover potential candidate disease miRNAs, we carried out our method on case studies of lung, breast, and pancreatic neoplasms. Because of space limitations, here, we focused on analyzing the candidates for lung neoplasms and listed the potential top 50 candidate miRNAs in detail (Table 4). For the other two diseases, we briefly analyzed the top 50 candidates, and their candidates are listed separately in Supplementary Table S1 and Supplementary Table S2, respectively. To ensure the reliability of prediction results, we first verified our predictions through four public databases, dbDEMC [38], PhenomiR [39], miRCancer [40], and TCGA [41]. Among them, dbDEMC explored miRNAs with abnormal expression in different cancers, where miRNAs with significantly different expression levels in cancer compared with normal tissues were retrieved and statistically analyzed through a “Significance Analysis of Microarrays” method. Similarly, PhenomiR consisted of dysregulated miRNAs associated with diseases. miRCancer provided a comprehensive collection of miRNA expression profiles in a variety of human cancers that are automatically extracted from published literature. TCGA sequenced the entire genome of some neoplasms, including at least 6000 candidate genes and microRNA sequences. It stored genomic characterization and sequence analysis of different tumor types. Since lung cancer is one of the most frequent cancers at present, we took lung neoplasms as an example and analyzed the top 50 candidate miRNAs in detail (Table 4). Among them, dbDMEC contained 43 candidates, and 32 candidates were verified by PhenomiR, indicating that they have been confirmed to be upregulated or downregulated in lung neoplasms. In addition, 10 candidates are included in the miRCancer, which further confirms their associations with the disease, and 7 miRNAs are contained in TCGA, indicating their different expression levels between cancer and normal tissues. The remaining 7 candidates were verified by the literature, where 5 miRNAs were confirmed to exert dysregulations in lung tissues compared with normal tissue [42,43,44,45,46]. miR-15a is involved in the regulation of non-small cell lung cancer and controls cell cycle progression in a synergistic and Rb-dependent manner [47], while miR-374a was confirmed to have different effects at different stages of lung cancer [48].

Among the top 50 candidates for breast neoplasms (ST1), dbDEMC and PhenomiR included 46 and 33 candidates, respectively, whose expression levels varied significantly in breast tumors compared with the normal tissues. The miRCancer contained 22 candidates indicating their associations with breast neoplasms, and 3 candidates were confirmed by TCGA, which demonstrates their different expression levels in different biological states. The remaining 3 candidates were verified by the literature. Among them, miR-142 is upregulated in human breast cancer stem cells (BCSCs) as compared to the non-tumorigenic breast cancer cells [49]. In addition, miR-542 can be used to predict the prognosis of breast cancer patients based on the mRNA expression of target gene lymphocyte antigen 9 (LY9), resulting in the secretion of frizzled protein-related protein 1 (SFRP1) [50]. miR-30e has separately been identified as an independent subtype-specific prognostic marker in breast cancer [51].

The top 50 pancreatic tumor candidates are listed in ST2, where 45 and 34 candidates are contained in the dbDEMC and PhenomiR, respectively. There are 19 candidates in the miRCancer that are known to be associated with the disease. Moreover, TCGA comprises 3 candidates. Five other candidates were also confirmed by the literature [52,53], where we also confirmed their different regulatory effects on pancreatic tumors. Moreover, the downregulation of the tumor protein UNC51-like kinase 1 (ULK1) by miR-372 inhibits the survival of human pancreatic cancer cells [54]. While miR-483 promotes cell proliferation by down-regulating its target gene Smad4 in pancreatic ductal adenocarcinoma (PDAC) cells. The three case studies provided above demonstrated the strong performance of CNNMDA in discovering potential disease associated miRNAs [55].

**Figure 3:**
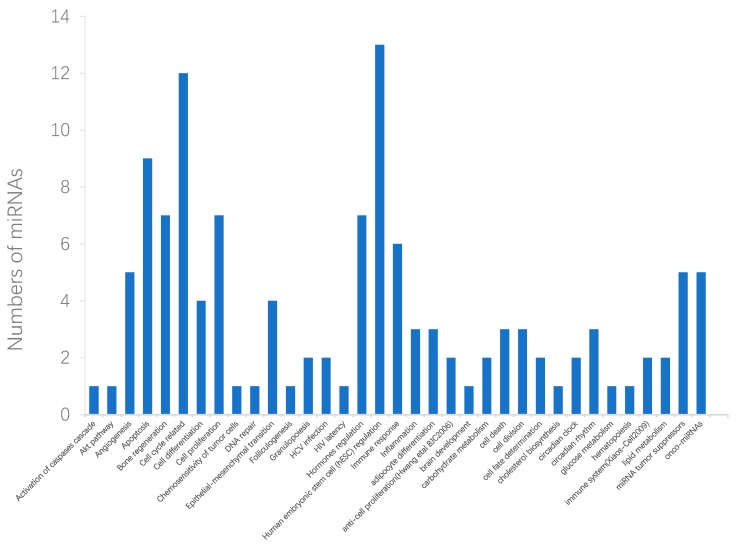
Functional enrichment analysis of lung cancer-related miRNAs. The horizontal ordinates represent 35 significant enriched functions of the top 50 candidate miRNAs associated with lung neoplasms. The vertical coordinates represent the number of miRNAs associated with each enriched function.

Functional enrichment analysis of miRNAs is helpful in understanding the function of disease-related miRNAs. Some tools [56,57,58] can be used to analyze the association between the function of the potential disease-associated miRNAs and disease progression. Among these tools, TAM [57] is a convenient online tool (http://cmbi.bjmu.edu.cn/tam), it integrates miRNAs into different sets according to various rules and provides investigators with the potential biological functions of the list of miRNAs. We performed functional enrichment analysis for the predicted top 50 potential disease-related miRNAs based on TAM. Here, we focused on the analysis of candidate miRNAs related to lung neoplasms (Figure 3). The results of the enrichment analysis of breast neoplasms and pancreatic neoplasms are listed in Supplementary Figures S1 and S2, respectively. Among the top 50 candidate miRNAs that relate to lung neoplasms, 12 miRNAs are involved in cell cycle-related functions, and 13 miRNAs are involved in human embryonic stem cell regulation functions. Furthermore, 9 miRNAs are concerned with apoptosis. In addition, 7, 7, and 6 miRNAs are related to cell proliferation, hormones regulation, and immune response, respectively. All the miRNA-related functions mentioned above have been confirmed to be closely related to the development of diseases. For instance, numerous studies have confirmed that cell cycle changes are closely related to cancer. When the normal cell cycle changes, the changes may lead to the division of some cells in the body and further cause cancer [59,60]. Specifically, it has been confirmed that cell cycle regulators play an important role in lung neoplasms [61]. As for human embryonic stem cell regulation, some research indicates it may be the origin of some solid tumors, including lung neoplasms, stomach neoplasms, and breast neoplasms [62,63]. Moreover, the metastasis of lung cancer may occur due to the dysregulation of some hormones in the human body [64], and the senescence of the immune system is a possible cause of lung cancer [65]. The other enriched functions associated with more miRNAs, such as apoptosis and cell proliferation, are related to the occurrence and development of diseases [66]. The above analysis can provide some insights into the putative roles of these candidates in lung neoplasms.

## 3. Materials and Methods

### 3.1. Dataset

We obtained miRNA-disease association data from the human miRNA-disease database (HMDD v2. 0) [67]. The database has collected thousands of miRNA-disease associations that have been experimentally verified. There were 492 miRNAs and 329 diseases in the dataset of our study, which contained 5218 known associations between them. The disease terms we used were derived from the U.S. National Library of Medicine. In terms of diseases, phenotype similarities and the semantic similarities between them were extracted from related literature [68].

### 3.2. Representation of miRNA and Disease Heterogeneous Data

#### 3.2.1. MiRNA Similarity Measure

miRNAs with approximate function have high probabilities of being associated with similar diseases. Most existing miRNA similarity data are obtained by calculating the similarity of the diseases to which they are associated. For example, miRNA $m_{1}$ is associated with diseases $d_{2}$, $d_{3}$, and $d_{4}$, miRNA $m_{2}$ is associated with diseases $d_{1}$, $d_{3}$, and $d_{4}$. By calculating the similarity between disease set {$d_{2}$, $d_{3}$, $d_{4}$} and set {$d_{1}$, $d_{3}$, $d_{4}$} as the similarity between $m_{1}$ and $m_{2}$ [69], it can be defined as $M_{12}$. miRNA similarities used in this study were calculated according to the above method. The similarity of $N_{m}$ miRNAs is represented by matrix $\left[M_{ij}\right]\in R^{N_{m}\times N_{m}}$ and each value is between 0 and 1.

#### 3.2.2. Disease Similarity Measure

Similarities between disease pairs can be judged by their semantics and phenotype; under normal conditions, if there are more common semantic terms and phenotypes between disease pairs, then they have a high probability of similarity. Accordingly, previous work calculated disease similarity based on the phenotypic and semantic information of the disease [29]. Disease similarities used in this study were obtained using Xuan’s method. The similarity of *N_d_* diseases are represented by matrix $\left[D_{ij}\right]\in R^{N_{d}\times N_{d}}$ and each value is also between 0 and 1.

#### 3.2.3. miRNA-Disease Associations

We used the matrix $A\in R^{N_{m}\times N_{m}}$ to represent the associations between $N_{m}$ miRNAs and $N_{d}$ diseases. If miRNA $m_{i}$ is known to be associated with a disease $d_{j}$, $A_{ij}=1$; contrastingly, $A_{ij}=0$ indicates that their association has not been explored.

### 3.3. Prediction Model Based on Network Representation Learning and Dual CNN

**Figure 4:**
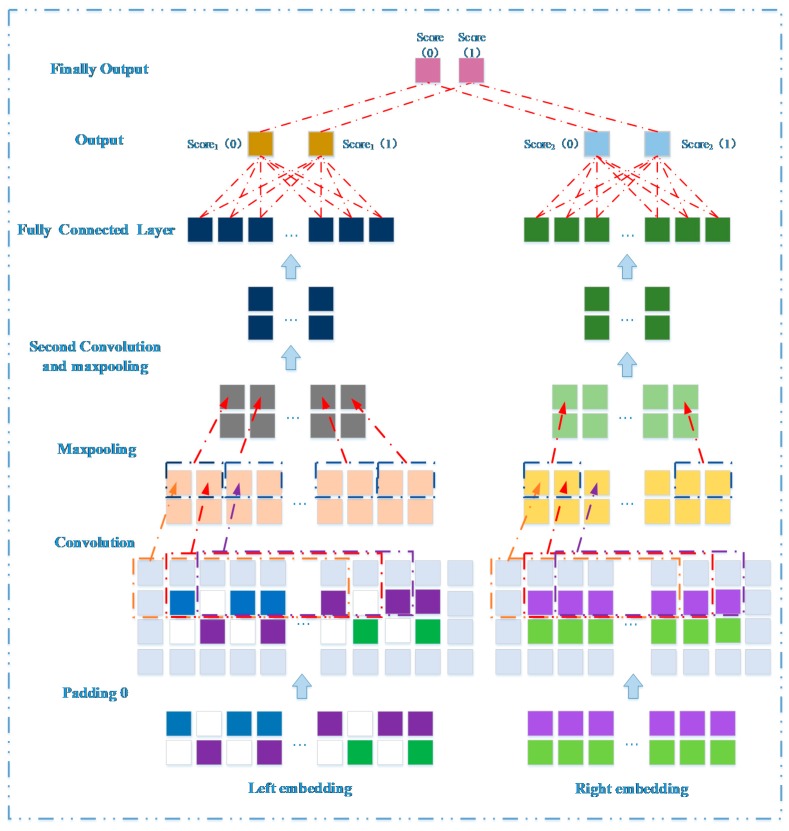
Construction of a deep learning framework based on dual convolutional neural networks to learn original representation and global network representation.

Here, we developed a novel prediction method based on network representation learning and dual CNN to infer potential miRNA-disease associations. Its prediction model is divided into a left part and a right part (Figure 4). The left part learns feature association representation between a miRNA $m_{i}$ and a disease $d_{j}$ through original feature information. The right part projects all miRNA and disease nodes into a low-dimensional space, thereby integrating their global information to obtain representative low-dimensional features of $m_{i}$ and $d_{j}$. These two parts use CNN layer deep learning node level representation and global level representation, respectively. Next, the two sides obtain prediction scores for $m_{i}$ and $d_{j}$ through the fully connected layer, respectively. Finally, we integrated two scores as a final prediction score between $m_{i}$ and $d_{j}$.

#### 3.3.1. Embedding Layer on the Left

**Figure 5:**
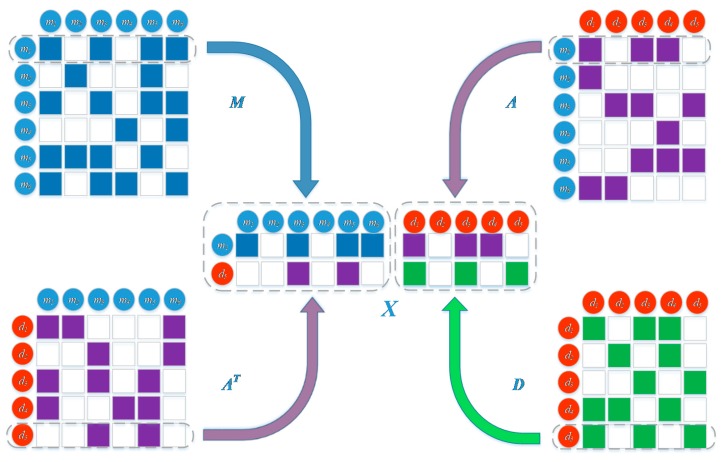
Establishment of the left embedding layer of miRNA *m*_1_ and disease *d*_5_ by combining their similarities and associations.

The left part integrates original feature information of miRNA and disease pairs. This is performed on the basis that miRNAs may be associated with similar diseases if they have similar functions and vice versa. Therefore, we combined miRNA and disease similarities as well as associations between them to form the feature representation of the left part. As an example, we have described the integration process of miRNA $m_{1}$ and disease $d_{5}$ (Figure 5). The first row of *M* is denoted as $M_{1}$. It contains similarity information between miRNA $m_{1}$ and all of the miRNAs. The fifth row of $A^{T}$ is denoted as $A_{5}^{T}$, it consists of the association of disease $d_{5}$ with all of the miRNAs. miRNA $m_{1}$ is similar to $m_{3}$, $m_{5}$, and $m_{6}$, and the disease $d_{5}$ has known association with $m_{3}$ and $m_{5}$. Thus $m_{1}$ and $d_{5}$ are likely to be associated, as they are all related to $m_{3}$ and $m_{5}$. Similarly, we integrate the first row of matrix *A* ($A_{1}$) together with the third row of matrix *D* ($D_{5}$). miRNA $m_{1}$ is known to be associated with $d_{1}$, $d_{3},$ and $d_{4}$, and disease $d_{5}$ is similar to $d_{1}$ and $d_{3}$, since both $m_{1}$ and $d_{5}$ are related to $d_{1}$ and $d_{3}$. Therefore $m_{1}$ and $d_{5}$ may be associated with each other. Finally, we integrated $M_{1}$, $A_{1}$, $D_{5}$, and $A_{5}^{T}$ to form the feature matrix $B\in R^{2\times \left(N_{m}+N_{d}\right)}$.

#### 3.3.2. Embedding Layer on the Right

In the right part, miRNA (disease) is projected into *k*-dimensional space to obtain representative low-dimensional features of miRNA and disease pairs, and integrate their global information. Non-negative matrix factorization (NMF) is an effective way to get a low-dimensional representation, and is widely used in data representation [70,71]. It aims to calculate two optimal non-negative matrices such that their product approximates the original matrix. Specifically, for the miRNA similarity matrix $M\in R^{N_{m}\times N_{m}}$, each row in it can be considered as a feature vector of a single miRNA, and we need to find non-negative matrices $W\in R^{N_{m}\times k}$ and $X\in R^{N_{m}\times k}$ whose products approximate to *M*, such as $M\approx WX^{T}$. Therefore, there is an optimization item as follows:

(3) $$\underset{W\geq 0,X\geq 0}{min}{\left\|M-WX^{T}\right\|}_{F}^{2},$$

where ${\left\|\cdot \right\|}_{F}$ is the Frobenius norm of a matrix, *X* represents a low-dimensional feature matrix of miRNA, and W is the basic matrix which is similar to the parameter matrix. Finally, *k* represents the target dimension that we reduce to.

Similarly, we also project disease information into *k*-dimensional space, in terms of disease similarity matrix $D\in R^{N_{d}\times N_{d}}$, calculating matrices $V\in R^{N_{d}\times k}$ and $Y\in R^{N_{d}\times k}$, and $D\approx VY^{T}$. Thus, combined with Equation (3), we obtain the following objective function:

(4) $$\underset{W,X,V,Y\geq 0}{min}{\left\|M-WX^{T}\right\|}_{F}^{2}+\alpha {\left\|D-VY^{T}\right\|}_{F}^{2},$$

where α is a parameter for control the contribution of the second item. *Y* represents a low-dimensional disease feature matrix, and *V* is a basic matrix.

The *i*-th row of feature matrix *X*, $x_{i}$, which is a row vector, represents the *k*-dimensional features of miRNA $m_{i}$. Similary, the *j*-th row of feature matrix *Y*, $y_{j}$, also a row vector, represents the *k*-dimensional features of disease $d_{j}$. If the *k*-dimensional features of $m_{i}$ and $d_{j}$ are mostly consistent, there may be potential links between them. The association probability between them is estimated by the formula $\left(x_{i}\right){\left(y^{T}\right)}_{j}={\left(xy^{T}\right)}_{ij}$, and the score should be close to $A_{ij}$, which is the true association probability between $m_{i}$ and $d_{j}$. As a result, we extend the objective function to:

(5) $$\underset{W,X,V,Y\geq 0}{min}{\left\|M-WX^{T}\right\|}_{F}^{2}+\alpha {\left\|D-VY^{T}\right\|}_{F}^{2}+\beta {\left\|A-XY^{T}\right\|}_{F}^{2},$$

where β is a parameter used to adjust the contribution of the third item.

In addition, if miRNA $m_{i}$ is similar to miRNA $m_{j}$, $m_{i}$ is likely related to other miRNAs whose similarity scores are relatively high with $m_{j}$. To preserve this network topology information, we introduce the graph regular term, which indicates that if the two miRNAs (diseases) $m_{i}$ and $m_{j}$ are close in original feature space, these two miRNAs (diseases) should also be closer to each other when their feature dimensions are reduced. However, prior to this, we need to establish a graph model for miRNA and disease feature matrices.

For the miRNA feature matrix, a graph model $S^{m}$ is constructed. The elements $S_{ij}^{m}$ are comprised of:

(6) $$S_{ij}^{m}=\{\begin{array}{ll} 1, & if\;m_{i}\;is\;the\;k-nearest\\ & \;neighbor\;of\;m_{j}\\ 0, & otherwise \end{array}$$

where $m_{i}$ and $m_{j}$ represent the *i*-th miRNA and the *j*-th miRNA, respectively. The similarity score between them is obtained from matrix M, and similarity scores of the $m_{i}$ are sorted with the rest of the miRNAs to determine whether $m_{j}$ belongs to the *k*-nearest of $m_{i}$.

For the disease feature matrix, a supplementary graph model $S^{d}$ is constructed:

(7) $$S_{pq}^{d}=\{\begin{array}{ll} 1, & if\;d_{p}\;is\;the\;k-nearest\\ & \;neighbor\;of\;d_{q}\\ 0, & otherwise \end{array}$$

where $d_{p}$ and $d_{q}$ represent disease *p* and disease *q*. The similarity between $d_{p}$ and $d_{q}$ are obtained from matrix *D*.

The graph regular terms for miRNAs and diseases are defined as:

(8) $$\frac{1}{2}\sum_{i,j=1}^{N_{m}}{\left\|x_{i}-x_{j}\right\|}^{2}S_{ij}^{m}=tr\left(X^{T}L_{m}X\right),$$

(9) $$\frac{1}{2}\sum_{p,q=1}^{N_{d}}{\left\|y_{p}-y_{q}\right\|}^{2}S_{pq}^{d}=tr\left(Y^{T}L_{d}Y\right),$$

where tr(.) represents the trace of a matrix, $x_{i}$ represents the *i*-th row of the matrix *X*, and $y_{p}$ represents the *p*-th row of the matrix Y. $L_{m}=D_{m}-S^{m}$ and $L_{d}=D_{d}-S^{d}$ are graph Laplacian matrices for $S^{m}$ and $S^{d}$, respectively, $D_{m}$ and $D_{d}$ are the diagonal matrices and $D_{m}\left(i,i\right)=\sum_{j=1}^{N_{m}}S^{m}\left(i,j\right)$, $D_{d}\left(p,p\right)=\sum_{q=1}^{N_{d}}S^{d}\left(p,q\right)$. Combining the graph regular terms into the objective function gives:

(10) $$\underset{W,X,V,Y\geq 0}{min}{\left\|M-WX^{T}\right\|}_{F}^{2}+\alpha {\left\|D-VY^{T}\right\|}_{F}^{2}+\beta {\left\|A-XY^{T}\right\|}_{F}^{2}\;+\lambda_{m}Tr\left(X^{T}L_{m}X\right)+\lambda_{d}Tr\left(Y^{T}L_{d}Y\right),$$

where $\lambda_{m}$ and $\lambda_{d}$ are parameters used to adjust the regularization terms.

Since the objective function in Equation (10) is not convex, it is unrealistic to hope to find a global optimal solution. We propose a strategy to find local minima by iteratively updating one item with other items fixed, such as updating *X* with *W*, *Y*, and *V* fixed. In addition, to constrain the matrix elements that are non-negative ($w_{ij}\geq 0,x_{ij}\geq 0,v_{pq}\geq 0,y_{pq}\geq 0$), we add the corresponding Lagrangian function. Finally, according to the trace and Frobenius norm of a matrix, the objective function *L* can also be expressed as:

(11) $$\begin{array}{cc} L= & Tr(MM^{T}-WX^{T}M^{T}-MXW^{T}+WX^{T}XW^{T})\\ & +\alpha Tr\left(DD^{T}-VY^{T}D^{T}-DYV^{T}+VY^{T}YV^{T}\right)\\ & +\beta Tr\left(AA^{T}-XY^{T}A^{T}-AYX^{T}+XY^{T}YX^{T}\right)\\ & +\lambda_{m}Tr\left(X^{T}L_{m}X\right)+\lambda_{d}Tr\left(Y^{T}L_{d}Y\right)\\ & +Tr\left(\delta W^{T}\right)+Tr\left(\mu X^{T}\right)+Tr\left(\phi V^{T}\right)+Tr\left(\theta Y^{T}\right), \end{array}$$

where δ, μ, φ, θ represents a Lagrange multiplier. Then the partial derivatives of *X*, *W*, *Y*, and *Z* can be calculated through the following function:

(12) $$\frac{\partial L}{\partial X}=-2M^{T}W+2XW^{T}W-2\beta AY+2\beta XY^{T}Y+2\lambda_{m}L_{m}X+\mu ,$$

(13) $$\frac{\partial L}{\partial W}=-2MX+2WX^{T}X+\delta ,$$

(14) $$\frac{\partial L}{\partial V}=-2\alpha DY+2\alpha VY^{T}Y+\phi ,$$

(15) $$\frac{\partial L}{\partial Y}=-2\alpha D^{T}V+2\alpha YV^{T}V-2\beta A^{T}X+2\beta YX^{T}X+2\lambda_{d}L_{d}Y+\theta .$$

According to Karush–Kuhn–Tucker (KKT) conditions [72], $\delta_{ij}w_{ij}=0$, $\mu_{ij}x_{ij}=0,\varphi_{ij}v_{ij}=0,\theta_{ij}y_{ij}=0$, the following equations are obtained:

(16) $${\left(-M^{T}W+XW^{T}W-\beta AY+\beta XY^{T}Y+\lambda_{m}L_{m}X\right)}_{ij}x_{ij}=0,$$

(17) $${\left(-MX+WX^{T}X\right)}_{ij}w_{ij}=0,$$

(18) $${\left(-\alpha DY+\alpha VY^{T}Y\right)}_{ij}v_{ij}=0,$$

(19) $${\left(-\alpha D^{T}V+\alpha YV^{T}V-\beta A^{T}X+\beta YX^{T}X+\lambda_{d}L_{d}Y\right)}_{ij}y_{ij}=0.$$

Finally, we obtained the following update rules:

(20) $$x_{ij}\leftarrow x_{ij}\frac{{\left(M^{T}W+\beta AY+\lambda_{m}S^{m}X\right)}_{ij}}{{\left(XW^{T}W+\beta XY^{T}Y+\lambda_{m}D_{m}X\right)}_{ij}},$$

(21) $$w_{ij}\leftarrow w_{ij}\frac{{\left(MX\right)}_{ij}}{{\left(WX^{T}X\right)}_{ij}},$$

(22) $$w_{ij}\leftarrow w_{ij}\frac{{\left(MX\right)}_{ij}}{{\left(WX^{T}X\right)}_{ij}},$$

(23) $$y_{ij}\leftarrow y_{ij}\frac{{\left(\alpha D^{T}V+\beta A^{T}X+\lambda_{d}S^{d}Y\right)}_{ij}}{{\left(\alpha YV^{T}V+\beta YX^{T}X+\lambda_{d}D_{d}Y\right)}_{ij}}.$$

**Figure 6:**
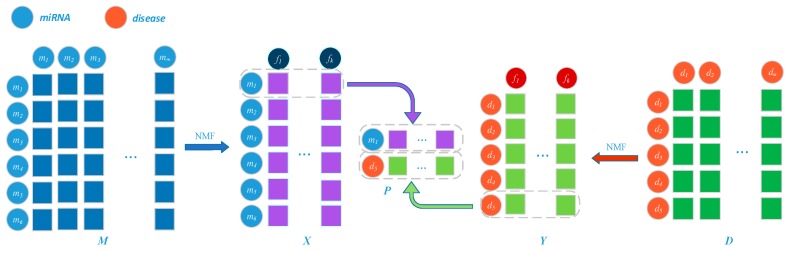
Establishment of the right embedding layer miRNA *m*_1_ and disease *d*_5_ by integrating their projection vectors in low-dimensional space.

Here, we iteratively update *W*, *X*, *V*, and *Y* through the above update formula until convergence. The first row of X, $x_{1}$, is the feature vector of miRNA $m_{1}$ and the fifth row of Y, $y_{5}$, is the feature vector of disease $d_{5}$. If the k-dimensional features of $m_{1}$ and $d_{5}$ are mostly consistent, there may be potential links between them. Moreover, $x_{1}$ and $y_{5}$ are integrated together to form a global feature representation matrix $P\;\epsilon \;R^{2\times k}$ (Figure 6).

#### 3.3.3. Convolutional Module on the Left

Feature matrix B, consisting of $m_{1}$ and $d_{5}$, is input to the CNN module to learn the original node pair representation between $m_{1}$ and $d_{5}$. In the convolutional layer, the convolution filter size is set to $w_{l}\times w_{f}$, and the number of filters is $n_{conv}$. Therefore, the convolution filters can be represented as $W_{conv}\in R^{w_{l}\times w_{f}\times n_{conv}}$. The output after the convolution operation is expressed as $C_{1}\in R^{2\times \left(N_{m}+N_{d}-w_{f}+1\right)\times n_{conv}}$. The following formulas represents the convolution process of *X*:

(24) $$X_{conv,i,j}=(X{\left(i,j,1\right)}_{,}X\left(i,j,2),\ldots ,X\left(i,j,j+w_{f}-1\right)\right)\;X_{conv,i,j}\in R^{w_{l}\times w_{f}},$$

(25) $$C_{1}\left(i,j,t\right)=g\left(X_{conv,i,j}*W_{conv}\left(:,:,t\right)+b_{conv}\left(t\right)\right),\;i\in \left[1,2\right],\;j\in \left[1,N_{m}+N_{d}-w_{f}+1\right],\;t\in \left[1,n_{conv}\right],$$

where X(i,j,1). indicates the first column vector in the sliding window when the filter moves to the *j*-th position of the *i*-th layer, and $C_{1}\left(i,j,t\right)$ represents the convolution result when the *t*-th filter slides to the *j*-th position of the *i*-th layer. *g* is a nonlinear activation function and $b_{conv}$ is a bias vector. In the above formula, the stride is set to 1 by default. In the pooling layer, we apply the max-pooling operation to compress the convolution result $C_{1}$, and get the output $P_{1}\in R^{\left(N_{m}+N_{d}\right)\times n_{conv}}$:

(26) $$P_{1}\left(i,p,t\right)=max(C_{1}\left(i,w_{p}*\left(p-1\right)+1,t\right),\ldots ,C_{1}\left(i,w_{p}*p,t\right)),$$

where $P_{1}\left(i,p,t\right)$ is the pooling result for the *p*-th position in the *i*-th row, and $w_{p}$ is the width of the sliding window in the pooling operation. Next, $P_{1}$ is used as the input to enter the second convolution layer after the same convolution and pooling operations as above to get the result $H_{1}\in R^{\frac{1}{2}\times \left(N_{m}+N_{d}\right)\times 2n_{conv}}$. We then flatten $H_{1}$ to a column vector $c\in R^{v\times 1}$($v=\frac{1}{2}\times \left(N_{m}+N_{d}\right)\times 2n_{conv}$). Finally, through the fully connected layer $W_{L}$ and the softmax layer, we obtain the association prediction score between $m_{1}$ and $d_{5}$. The score is defined as $score_{1}\in R^{2\times 1}$:

(27) $$score_{1}=W_{L}\times c.$$

#### 3.3.4. Convolutional Module on the Right

The embedding in the right part, $P\in R^{2\times k}$, is used as input to learn global information about miRNA $m_{1}$ and disease $d_{5}$ through their representative *k*-dimensional features. The process of convolution and pooling on the right is similar to the left, and the detailed operation process is defined as follows:

(28) $$Y_{conv,i,j}=(Y{\left(i,j,1\right)}_{,}Y\left(i,j,2),\ldots ,Y\left(i,j,j+w_{f}-1\right)\right)\;Y_{conv,i,j}\in R^{w_{l}\times w_{f}},$$

(29) $$C_{2}\left(i,j,t\right)=g\left(Y_{conv,i,j}*W_{conv}\left(:,:,t\right)+b_{conv}\left(t\right)\right),$$

(30) $$P_{2}\left(i,p,t\right)=max(C_{2}\left(i,w_{p}*\left(p-1\right)+1,t\right),\ldots ,C_{2}\left(i,w_{p}*p,t\right)),$$

where *Y* indicates the value of the sliding window at different positions. $C_{2}$ is the feature output after the convolution layer, which then passes through the pooling layer to obtain $P_{2}$. We also use $P_{2}$ as the input for the next convolution layer, and obtain the output $H_{2}\in R^{\frac{1}{2}\times k\times 2n_{conv}}$ through convolution and pooling operations. The next step is to flatten $H_{2}$ to a column vector $o\in R^{v\times 1}$($v=\frac{1}{2}\times k\times 2n_{conv}$). Finally, through the fully connected layer $W_{R}$ and the softmax layer, we obtain the association prediction score between $m_{1}$ and $d_{5}$. The score is defined as $score_{2}\in R^{2\times 1}$:

(31) $$score_{2}=W_{R}\times o.$$

#### 3.3.5. Combined Strategy

Considering the two parts of the prediction scores between $m_{1}$ and $d_{5}$ from different perspectives, the optimal performance of the two parts may be different. Therefore, we integrated $score_{1}$ and $score_{2}$ as the final association score. It is defined as follows:

(32) $$score=\lambda \times score_{1}+\left(1-\lambda \right)\times score_{2},$$

where λ∈(0,1) is a parameter used to weigh the score contributions of $score_{1}$ and $score_{2}$. The left and right CNN models all establish a loss function based on cross entropy, defined as $loss_{1}$ and $loss_{2}$, respectively:

(33) $$loss_{1}=-\sum_{i=1}^{T}\left[y_{label}\times log\;a+\left(1-y_{label}\right)\times log(1-a\right)],$$

(34) $$a=\frac{e^{score_{1}\left(1\right)}}{e^{score_{1}\left(0\right)}+e^{score_{1}\left(1\right)}},$$

(35) $$loss_{2}=-\sum_{i=1}^{T}\left[y_{label}\times log\;b+\left(1-y_{label}\right)\times log(1-b\right)],$$

(36) $$b=\frac{e^{score_{2}\left(1\right)}}{e^{score_{2}\left(0\right)}+e^{score_{2}\left(1\right)}},$$

where $y_{lable}$ represents the actual associated label between the miRNA and the disease. If the association between the miRNA and the disease is known, $y_{lable}=1$, otherwise, $y_{lable}=0$. $score_{1}\left(0\right)$ and $score_{1}\left(1\right)$ represent the association scores of miRNAs and diseases on the left side. It is similar to a binary classification problem, where $score_{1}\left(0\right)$ represents the probability that $m_{1}$ and $d_{5}$ are not associated, and $score_{1}\left(1\right)$ represents the probability of an association. Finally, we used the softmax function to obtain the association probability *a*. Similarly, for the calculated right path association probability *b*, score(1) indicates the final prediction score between $m_{1}$ and $d_{5}$, and *T* represents the number of training samples.

### 3.4. Predicting Novel Disease-Related miRNAs

The predictive performance of CNNMDA was evaluated through a cross-validation process and several case studies, and was applied to predict potential candidate miRNAs for all 329 diseases. We used all positive and negative samples to train CNNMDA. The predicted results of 329 diseases are listed in Supplementary Table S3. Moreover, the candidate miRNAs related to 3 diseases are analyzed in case studies and they come from Supplementary Table S3.

## 4. Conclusions

CNNMDA has been developed as a novel method based on network representation learning and dual convolutional neural networks for predicting potential miRNA-disease associations. CNNMDA captures the internal relationships between miRNAs and diseases, including miRNA similarities and disease similarities. Meanwhile, it also captures the associations between miRNAs and diseases. Moreover, the representations of the miRNA nodes and the disease nodes are learned based on an entire miRNA-disease network, and as such are deeply integrated to enhance logical reasoning. The new framework based on network representation learning and dual convolutional neural networks is able to learn the original and global representations of a miRNA-disease pair. CNNMDA’s performance was verified by cross-validation with 15 common diseases and case studies on 3 diseases. Experimental results indicated that CNNMDA outperforms existing methods in terms of both AUCs and AUPRs. It is able to generate reliable candidate miRNA-disease associations for subsequent validation by biologists.

## Figures and Tables

**Table 1:** Prediction results of CNNMDA and the other four methods for 15 diseases in terms of the area under the receiver operating characteristic curve (AUC).

Diseases Name	AUC CNNMDA	GSTRW	DMPred	BNPMDA	Liu’s Method
Breast neoplasms	**0.991**	0.822	0.939	0.906	0.896
Hepatocellular carcinoma	**0.978**	0.770	0.899	0.784	0.846
Renal cell carcinoma	**0.960**	0.801	0.897	0.830	0.785
Squamous cell carcinoma	**0.932**	0.821	0.894	0.793	0.897
Colorectal neoplasms	**0.924**	0.742	0.882	0.724	0.864
Glioblastoma	**0.916**	0.821	0.906	0.781	0.828
Heart failure	**0.986**	0.823	0.984	0.929	0.816
Acute myeloid leukemia	**0.969**	0.817	0.894	0.784	0.924
Lung neoplasms	**0.987**	0.795	0.941	0.903	0.931
Melanoma	**0.994**	0.788	0.909	0.909	0.859
Ovarian neoplasms	**0.955**	0.831	0.934	0.924	0.855
Pancreatic neoplasms	**0.971**	0.853	0.913	0.725	0.892
Prostatic neoplasms	**0.982**	0.828	0.947	0.896	0.895
Stomach neoplasms	**0.994**	0.781	0.922	0.740	0.838
Urinary bladder neoplasms	**0.982**	0.821	0.921	0.879	0.870

The bold values indicate the higher AUCs.

**Table 2:** Prediction results of CNNMDA and other four methods for 15 diseases in terms of the area under the precision–recall curve (AUPR).

Diseases Name	AUPR CNNMDA	GSTRW	DMPred	BNPMDA	Liu’s Method
Breast neoplasms	**0.919**	0.261	0.681	0.245	0.378
Hepatocellular carcinoma	**0.871**	0.234	0.539	0.574	0.335
Renal cell carcinoma	**0.549**	0.127	0.325	0.328	0.152
Squamous cell carcinoma	**0.290**	0.104	0.191	0.272	0.170
Colorectal neoplasms	**0.425**	0.136	0.279	0.177	0.273
Glioblastoma	0.277	0.142	0.270	**0.452**	0.166
Heart failure	**0.874**	0.160	0.669	0.451	0.157
Acute myeloid leukemia	0.262	0.118	0.236	**0.367**	0.207
Lung neoplasms	**0.706**	0.140	0.481	0.480	0.343
Melanoma	**0.896**	0.157	0.410	0.477	0.309
Ovarian neoplasms	**0.543**	0.152	0.453	0.386	0.239
Pancreatic neoplasms	**0.593**	0.133	0.308	0.136	0.283
Prostatic neoplasms	**0.673**	0.150	0.414	0.175	0.231
Stomach neoplasms	**0.881**	0.207	0.503	0.306	0.303
Urinary bladder neoplasms	**0.694**	0.134	0.331	0.292	0.229

The bold values indicate the higher AUPRs.

**Table 3:** Comparison of different methods based on AUCs with a paired *t*-test.

*p*-Value between CNNMDA and Another Method	DMPred	GSTRW	BNPMDA	Liu’s Method
*p*-values of ROC curves	3.3219 × 10^−5^	8.5916 × 10^−23^	5.4483 × 10^−10^	2.0247 × 10^−10^
*p*-values of PR curves	1.4386 × 10^−8^	2.7951 × 10^−13^	1.181 × 10^−2^	2.9012 × 10^−8^

**Table 4:** The top 50 lung neoplasms-related candidates.

Rank	miRNA Name	Evidence
1	hsa-mir-106b	dbDEMC, PhenomiR
2	hsa-mir-15a	Literature [47]
3	hsa-mir-16	dbDEMC, PhenomiR, miRCancer
4	hsa-mir-130a	dbDEMC, PhenomiR
5	hsa-mir-193b	dbDEMC, PhenomiR, TCGA
6	hsa-mir-520d	dbDEMC
7	hsa-mir-429	dbDEMC, miRCancer
8	hsa-mir-122	dbDEMC, PhenomiR, miRCancer
9	hsa-mir-149	dbDEMC, PhenomiR
10	hsa-mir-424	dbDEMC, PhenomiR
11	hsa-mir-451a	dbDEMC
12	hsa-mir-378a	Literature [42]
13	hsa-mir-708	dbDEMC
14	hsa-mir-20b	dbDEMC, PhenomiR, TCGA
15	hsa-mir-15b	dbDEMC, PhenomiR, miRCancer
16	hsa-mir-520a	dbDEMC, TCGA
17	hsa-mir-10a	dbDEMC
18	hsa-mir-520b	dbDEMC
19	hsa-mir-625	dbDEMC
20	hsa-mir-141	dbDEMC, PhenomiR, miRCancer
21	hsa-mir-449a	dbDEMC, PhenomiR, miRCancer
22	hsa-mir-99a	dbDEMC, PhenomiR, TCGA
23	hsa-mir-195	dbDEMC, PhenomiR, miRCancer
24	hsa-mir-151a	Literature [43]
25	hsa-mir-296	Literature [44]
26	hsa-mir-449b	dbDEMC, PhenomiR, miRCancer
27	hsa-mir-28	dbDEMC, PhenomiR
28	hsa-mir-342	dbDEMC, PhenomiR
29	hsa-mir-372	dbDEMC, PhenomiR, TCGA
30	hsa-mir-345	dbDEMC, PhenomiR
31	hsa-mir-92b	dbDEMC, PhenomiR
32	hsa-mir-328	dbDEMC, PhenomiR
33	hsa-mir-367	dbDEMC, PhenomiR
34	hsa-mir-373	dbDEMC, PhenomiR
35	hsa-mir-302b	dbDEMC, PhenomiR, miRCancer
36	hsa-mir-194	dbDEMC, PhenomiR
37	hsa-mir-1258	dbDEMC
38	hsa-mir-320a	dbDEMC, PhenomiR
39	hsa-mir-152	dbDEMC, PhenomiR
40	hsa-mir-302c	dbDEMC, PhenomiR
41	hsa-mir-151b	dbDEMC
42	hsa-mir-204	dbDEMC, PhenomiR
43	hsa-mir-23b	dbDEMC, PhenomiR
44	hsa-mir-129	dbDEMC, PhenomiR, TCGA
45	hsa-mir-451b	Literature [45]
46	hsa-mir-374a	Literature [48]
47	hsa-mir-211	dbDEMC, PhenomiR
48	hsa-mir-208a	Literature [46]
49	hsa-mir-1254	dbDEMC, miRCancer
50	hsa-mir-337	dbDEMC, PhenomiR, TCGA

## Supplementary Materials

Supplementary materials can be found at https://www.mdpi.com/1422-0067/20/15/3648/s1.

## References

[1] Chen K., Rajewsky N. (2007). The evolution of gene regulation by transcription factors and microRNAs. Nat. Rev. Genet..

[2] Subramanian S., Fu Y., Sunkar R., Barbazuk W.B., Zhu J.-K., Yu O. (2008). Novel and nodulation-regulated microRNAs in soybean roots. BMC Genom..

[3] Zhang B., Wang Q., Pan X. (2007). MicroRNAs and their regulatory roles in animals and plants. J. Cell. Physiol..

[4] Calin G.A., Croce C.M. (2006). MicroRNA signatures in human cancers. Nat. Rev. Cancer.

[5] Chen X., Xie D., Zhao Q., You Z.-H. (2017). MicroRNAs and complex diseases: From experimental results to computational models. Brief. Bioinform..

[6] Gaur A., Jewell D.A., Liang Y., Ridzon D., Moore J.H., Chen C., Ambros V.R., Israel M.A. (2007). Characterization of microRNA expression levels and their biological correlates in human cancer cell lines. Cancer Res..

[7] Meola N., Gennarino V.A., Banfi S. (2009). microRNAs and genetic diseases. Pathogenetics.

[8] Bartel D.P. (2004). MicroRNAs: Genomics, biogenesis, mechanism, and function. Cell.

[9] Jiang Q., Hao Y., Wang G., Juan L., Zhang T., Teng M., Liu Y., Wang Y. (2010). Prioritization of disease microRNAs through a human phenome-microRNAome network. BMC Syst. Biol..

[10] Qabaja A., Alshalalfa M., Bismar T.A., Alhajj R. (2013). Protein network-based Lasso regression model for the construction of disease-miRNA functional interactions. EURASIP J. Bioinform. Syst. Biol..

[11] Shi H., Xu J., Zhang G., Xu L., Li C., Wang L., Zhao Z., Jiang W., Guo Z., Li X. (2013). Walking the interactome to identify human miRNA-disease associations through the functional link between miRNA targets and disease genes. BMC Syst. Biol..

[12] Xu C., Ping Y., Li X., Zhao H., Wang L., Fan H., Xiao Y., Li X. (2014). Prioritizing candidate disease miRNAs by integrating phenotype associations of multiple diseases with matched miRNA and mRNA expression profiles. Mol. Biosyst..

[13] Kertesz M., Iovino N., Unnerstall U., Gaul U., Segal E. (2007). The role of site accessibility in microRNA target recognition. Nat. Genet..

[14] Lewis B.P., Shih I.-h., Jones-Rhoades M.W., Bartel D.P., Burge C.B. (2003). Prediction of mammalian microRNA targets. Cell.

[15] Bandyopadhyay S., Mitra R., Maulik U., Zhang M.Q. (2010). Development of the human cancer microRNA network. Silence.

[16] Barabási A.-L., Gulbahce N., Loscalzo J. (2011). Network medicine: A network-based approach to human disease. Nat. Rev. Genet..

[17] Paci P., Colombo T., Fiscon G., Gurtner A., Pavesi G., Farina L. (2017). SWIM: A computational tool to unveiling crucial nodes in complex biological networks. Sci. Rep..

[18] Fiscon G., Conte F., Farina L., Paci P. (2018). Network-based approaches to explore complex biological systems towards network medicine. Genes.

[19] Fiscon G., Conte F., Farina L., Pellegrini M., Russo F., Paci P. (2019). Identification of Disease–miRNA Networks Across Different Cancer Types Using SWIM. MicroRNA Target Identification.

[20] Xu J., Li C.-X., Lv J.-Y., Li Y.-S., Xiao Y., Shao T.-T., Huo X., Li X., Zou Y., Han Q.-L. (2011). Prioritizing candidate disease miRNAs by topological features in the miRNA target–dysregulated network: Case study of prostate cancer. Mol. Cancer Ther..

[21] Chen X., Liu M.-X., Yan G.-Y. (2012). RWRMDA: Predicting novel human microRNA–disease associations. Mol. BioSyst..

[22] Xuan P., Han K., Guo Y., Li J., Li X., Zhong Y., Zhang Z., Ding J. (2015). Prediction of potential disease-associated microRNAs based on random walk. Bioinformatics.

[23] Liu Y., Zeng X., He Z., Zou Q. (2016). Inferring microRNA-disease associations by random walk on a heterogeneous network with multiple data sources. IEEE/ACM Trans. Comput. Biol. Bioinform..

[24] Luo J., Xiao Q. (2017). A novel approach for predicting microRNA-disease associations by unbalanced bi-random walk on heterogeneous network. J. Biomed. Inform..

[25] Chen X., Huang L. (2017). LRSSLMDA: Laplacian regularized sparse subspace learning for MiRNA-disease association prediction. PLoS Comput. Biol..

[26] Shen Z., Zhang Y.-H., Han K., Nandi A.K., Honig B., Huang D.-S. (2017). miRNA-disease association prediction with collaborative matrix factorization. Complexity.

[27] Xiao Q., Luo J., Liang C., Cai J., Ding P. (2017). A graph regularized non-negative matrix factorization method for identifying microRNA-disease associations. Bioinformatics.

[28] Xuan P., Shen T., Wang X., Zhang T., Zhang W. (2018). Inferring disease-associated microRNAs in heterogeneous networks with node attributes. IEEE/ACM Trans. Comput. Biol. Bioinform..

[29] Zhong Y., Xuan P., Wang X., Zhang T., Li J., Liu Y., Zhang W. (2017). A non-negative matrix factorization based method for predicting disease-associated miRNAs in miRNA-disease bilayer network. Bioinformatics.

[30] Zeng X., Liu L., Lü L., Zou Q. (2018). Prediction of potential disease-associated microRNAs using structural perturbation method. Bioinformatics.

[31] Luo J., Ding P., Liang C., Cao B., Chen X. (2016). Collective prediction of disease-associated miRNAs based on transduction learning. IEEE/ACM Trans. Comput. Biol. Bioinform..

[32] Chen X., Wang L., Qu J., Guan N.-N., Li J.-Q. (2018). Predicting miRNA–disease association based on inductive matrix completion. Bioinformatics.

[33] Chen X., Xie D., Wang L., Zhao Q., You Z.-H., Liu H. (2018). BNPMDA: Bipartite network projection for MiRNA–disease association prediction. Bioinformatics.

[34] Che K., Guo M., Wang C., Liu X., Chen X. (2019). Predicting MiRNA-Disease Association by Latent Feature Extraction with Positive Samples. Genes.

[35] Saito T., Rehmsmeier M. (2015). The precision-recall plot is more informative than the ROC plot when evaluating binary classifiers on imbalanced datasets. PLoS ONE.

[36] Xuan P., Sun C., Zhang T., Ye Y., Shen T., Dong Y. (2019). Gradient Boosting Decision Tree-Based Method for Predicting Interactions Between Target Genes and Drugs. Front. Genet..

[37] Chen M., Liao B., Li Z. (2018). Global Similarity Method Based on a Two-tier Random Walk for the Prediction of microRNA–Disease Association. Sci. Rep..

[38] Yang Z., Ren F., Liu C., He S., Sun G., Gao Q., Yao L., Zhang Y., Miao R., Cao Y. (2010). dbDEMC: A database of differentially expressed miRNAs in human cancers. BMC Genom..

[39] Ruepp A., Kowarsch A., Schmidl D., Buggenthin F., Brauner B., Dunger I., Fobo G., Frishman G., Montrone C., Theis F.J. (2010). PhenomiR: A knowledgebase for microRNA expression in diseases and biological processes. Genome Biol..

[40] Xie B., Ding Q., Han H., Wu D. (2013). miRCancer: A microRNA–cancer association database constructed by text mining on literature. Bioinformatics.

[41] Tomczak K., Czerwińska P., Wiznerowicz M. (2015). The Cancer Genome Atlas (TCGA): An immeasurable source of knowledge. Contemp. Oncol..

[42] Chen L.-T., Xu S.-D., Xu H., Zhang J.-F., Ning J.-F., Wang S.-F. (2012). MicroRNA-378 is associated with non-small cell lung cancer brain metastasis by promoting cell migration, invasion and tumor angiogenesis. Med. Oncol..

[43] Daugaard I., Sanders K., Idica A., Vittayarukskul K., Hamdorf M., Krog J., Chow R., Jury D., Hansen L., Hager H. (2017). miR-151a induces partial EMT by regulating E-cadherin in NSCLC cells. Oncogenesis.

[44] Hu L., Ai J., Long H., Liu W., Wang X., Zuo Y., Li Y., Wu Q., Deng Y. (2016). Integrative microRNA and gene profiling data analysis reveals novel biomarkers and mechanisms for lung cancer. Oncotarget.

[45] Shen W., Liu J., Zhao G., Fan M., Song G., Zhang Y., Weng Z., Zhang Y. (2017). Repression of Toll-like receptor-4 by microRNA-149-3p is associated with smoking-related COPD. Int. J. Chronic Obstr. Pulm. Dis..

[46] Tang Y., Cui Y., Li Z., Jiao Z., Zhang Y., He Y., Chen G., Zhou Q., Wang W., Zhou X. (2016). Radiation-induced miR-208a increases the proliferation and radioresistance by targeting p21 in human lung cancer cells. J. Exp. Clin. Cancer Res..

[47] Bandi N., Vassella E. (2011). miR-34a and miR-15a/16 are co-regulated in non-small cell lung cancer and control cell cycle progression in a synergistic and Rb-dependent manner. Mol. Cancer.

[48] Zhao M., Xu P., Liu Z., Zhen Y., Chen Y., Liu Y., Fu Q., Deng X., Liang Z., Li Y. (2018). Dual roles of miR-374a by modulated c-Jun respectively targets CCND1-inducing PI3K/AKT signal and PTEN-suppressing Wnt/β-catenin signaling in non-small-cell lung cancer. Cell Death Dis..

[49] Isobe T., Hisamori S., Hogan D.J., Zabala M., Hendrickson D.G., Dalerba P., Cai S., Scheeren F., Kuo A.H., Sikandar S.S. (2014). miR-142 regulates the tumorigenicity of human breast cancer stem cells through the canonical WNT signaling pathway. Elife.

[50] Zhu Q.-N., Renaud H., Guo Y. (2018). Bioinformatics-based identification of miR-542-5p as a predictive biomarker in breast cancer therapy. Hereditas.

[51] D’aiuto F., Callari M., Dugo M., Merlino G., Musella V., Miodini P., Paolini B., Cappelletti V., Daidone M. (2015). miR-30e* is an independent subtype-specific prognostic marker in breast cancer. Br. J. Cancer.

[52] Gui Z., Li S., Liu X., Xu B., Xu J. (2015). Oridonin alters the expression profiles of microRNAs in BxPC-3 human pancreatic cancer cells. BMC Complement. Altern. Med..

[53] Yu J., Li A., Hong S.-M., Hruban R.H., Goggins M. (2012). MicroRNA alterations of pancreatic intraepithelial neoplasias. Clin. Cancer Res..

[54] Chen H., Zhang Z., Lu Y., Song K., Liu X., Xia F., Sun W. (2017). Downregulation of ULK 1 by micro RNA-372 inhibits the survival of human pancreatic adenocarcinoma cells. Cancer Sci..

[55] Hao J., Zhang S., Zhou Y., Hu X., Shao C. (2011). MicroRNA 483-3p suppresses the expression of DPC4/Smad4 in pancreatic cancer. FEBS Lett..

[56] Backes C., Khaleeq Q.T., Meese E., Keller A. (2016). miEAA: microRNA enrichment analysis and annotation. Nucleic Acids Res..

[57] Li J., Han X., Wan Y., Zhang S., Zhao Y., Fan R., Cui Q., Zhou Y. (2018). TAM 2.0: Tool for MicroRNA set analysis. Nucleic Acids Res..

[58] Fan Y., Habib M., Xia J. (2018). Xeno-miRNet: A comprehensive database and analytics platform to explore xeno-miRNAs and their potential targets. PeerJ.

[59] Park M.-T., Lee S.-J. (2003). Cell cycle and cancer. J. Biochem. Mol. Biol..

[60] Collins K., Jacks T., Pavletich N.P. (1997). The cell cycle and cancer. Proc. Natl. Acad. Sci. USA.

[61] Eymin B., Gazzeri S. (2010). Role of cell cycle regulators in lung carcinogenesis. Cell Adhes. Migr..

[62] Visvader J.E. (2011). Cells of origin in cancer. Nature.

[63] Martin-Belmonte F., Perez-Moreno M. (2012). Epithelial cell polarity, stem cells and cancer. Nat. Rev. Cancer.

[64] Deng X., Tannehill-Gregg S.H., Nadella M.V., He G., Levine A., Cao Y., Rosol T.J. (2007). Parathyroid hormone-related protein and ezrin are up-regulated in human lung cancer bone metastases. Clin. Exp. Metastasis.

[65] Domagala-Kulawik J., Osinska I., Hoser G. (2014). Mechanisms of immune response regulation in lung cancer. Transl. Lung Cancer Res..

[66] Liu G., Pei F., Yang F., Li L., Amin A., Liu S., Buchan J., Cho W. (2017). Role of autophagy and apoptosis in non-small-cell lung cancer. Int. J. Mol. Sci..

[67] Li Y., Qiu C., Tu J., Geng B., Yang J., Jiang T., Cui Q. (2013). HMDD v2. 0: A database for experimentally supported human microRNA and disease associations. Nucleic Acids Res..

[68] Hoehndorf R., Schofield P.N., Gkoutos G.V. (2015). The role of ontologies in biological and biomedical research: A functional perspective. Brief. Bioinform..

[69] Wang D., Wang J., Lu M., Song F., Cui Q. (2010). Inferring the human microRNA functional similarity and functional network based on microRNA-associated diseases. Bioinformatics.

[70] Hosoda K., Watanabe M., Wersing H., Körner E., Tsujino H., Tamura H., Fujita I. (2009). A model for learning topographically organized parts-based representations of objects in visual cortex: Topographic nonnegative matrix factorization. Neural Comput..

[71] Zheng C.-H., Huang D.-S., Zhang L., Kong X.-Z. (2009). Tumor clustering using nonnegative matrix factorization with gene selection. IEEE Trans. Inf. Technol. Biomed..

[72] Facchinei F., Kanzow C., Sagratella S. (2014). Solving quasi-variational inequalities via their KKT conditions. Math. Program..

