# Supplementary material for: Inferring the Disease-Associated miRNAs Based on Network Representation Learning and Convolutional Neural Networks
Source: Int J Mol Sci. 2019 Jul 25;20(15):3648. doi: 10.3390/ijms20153648 (PMC6696449; doi:10.3390/ijms20153648)
Supplement: Supplementary file 1 [file ijms-20-03648-s001.zip › ST1_breast neoplasms.pdf]

Supplementary Table S1 the top 50 breast neoplasms -related miRNA candidates

Rank	MiRNA Name	Evidence
1	hsa-mir-99a	dbDEMC, PhenomiR, miRCancer
2	hsa-mir-106a	dbDEMC
3	hsa-mir-372	dbDEMC, PhenomiR
4	hsa-mir-185	dbDEMC, PhenomiR, miRCancer
5	hsa-mir-138	dbDEMC, PhenomiR, miRCancer
6	hsa-mir-150	dbDEMC, PhenomiR, miRCancer
7	hsa-mir-378a	TCGA
8	hsa-mir-142	Literature
9	hsa-mir-192	dbDEMC, PhenomiR
10	hsa-mir-15b	dbDEMC, PhenomiR
11	hsa-mir-92b	dbDEMC
12	hsa-mir-542	Literature
13	hsa-mir-98	dbDEMC, PhenomiR, miRCancer
14	hsa-mir-95	dbDEMC, PhenomiR
15	hsa-mir-184	dbDEMC, PhenomiR
16	hsa-mir-449a	dbDEMC, PhenomiR, miRCancer
17	hsa-mir-196b	dbDEMC, PhenomiR
18	hsa-mir-186	dbDEMC, PhenomiR
19	hsa-mir-130a	dbDEMC, PhenomiR, miRCancer
20	hsa-mir-449b	dbDEMC
21	hsa-mir-99b	dbDEMC, PhenomiR
22	hsa-mir-28	dbDEMC, PhenomiR
23	hsa-mir-130b	dbDEMC, PhenomiR
24	hsa-mir-212	dbDEMC, PhenomiR, miRCancer
25	hsa-mir-1254	dbDEMC
26	hsa-mir-1224	dbDEMC
27	hsa-mir-494	dbDEMC, PhenomiR, miRCancer, TCGA
28	hsa-mir-32	dbDEMC, PhenomiR, miRCancer
29	hsa-mir-330	dbDEMC, PhenomiR, miRCancer
30	hsa-mir-744	dbDEMC
31	hsa-mir-211	dbDEMC, PhenomiR, miRCancer
32	hsa-mir-302f	dbDEMC
33	hsa-mir-384	dbDEMC, miRCancer
34	hsa-mir-181c	dbDEMC, PhenomiR
35	hsa-mir-498	dbDEMC, PhenomiR, miRCancer
36	hsa-mir-30e	Literature
37	hsa-mir-363	dbDEMC
38	hsa-mir-421	dbDEMC, miRCancer
39	hsa-mir-302e	dbDEMC
40	hsa-mir-208a	dbDEMC, PhenomiR
41	hsa-mir-216b	dbDEMC, miRCancer, TCGA

42	hsa-mir-216a	dbDEMC, PhenomiR
43	hsa-mir-198	dbDEMC, PhenomiR, miRCancer
44	hsa-mir-33b	dbDEMC, PhenomiR, miRCancer
45	hsa-mir-181d	dbDEMC, PhenomiR
46	hsa-mir-144	dbDEMC, PhenomiR, miRCancer
47	hsa-mir-33a	dbDEMC, PhenomiR, miRCancer
48	hsa-mir-433	dbDEMC, PhenomiR, miRCancer
49	hsa-mir-1271	dbDEMC, miRCancer
50	hsa-mir-663b	dbDEMC, PhenomiR
