# Supplementary material for: Inferring the Disease-Associated miRNAs Based on Network Representation Learning and Convolutional Neural Networks
Source: Int J Mol Sci. 2019 Jul 25;20(15):3648. doi: 10.3390/ijms20153648 (PMC6696449; doi:10.3390/ijms20153648)
Supplement: Supplementary file 1 [file ijms-20-03648-s001.zip › ST2_pancreatic neoplasms.pdf]

Supplementary Table S2 the top 50 pancreatic neoplasms-related miRNA candidates

Rank	MiRNA Name	Evidence
1	hsa-mir-9	dbDEMC, PhenomiR, miRCancer
2	hsa-mir-106b	dbDEMC, PhenomiR
3	hsa-mir-1	dbDEMC, PhenomiR, miRCancer
4	hsa-mir-29a	dbDEMC, PhenomiR, miRCancer
5	hsa-mir-181a	dbDEMC, PhenomiR, miRCancer
6	hsa-mir-130a	dbDEMC, PhenomiR
7	hsa-mir-7	dbDEMC, PhenomiR, miRCancer
8	hsa-mir-19a	dbDEMC
9	hsa-mir-22	dbDEMC, PhenomiR
10	hsa-mir-125a	dbDEMC, PhenomiR
11	hsa-mir-30a	dbDEMC, PhenomiR
12	hsa-mir-29c	dbDEMC, PhenomiR, miRCancer
13	hsa-mir-20b	dbDEMC
14	hsa-mir-141	dbDEMC, PhenomiR, miRCancer
15	hsa-mir-133a	dbDEMC, PhenomiR, miRCancer, TCGA
16	hsa-mir-205	dbDEMC, PhenomiR, miRCancer
17	hsa-mir-19b	dbDEMC, TCGA
18	hsa-mir-193b	dbDEMC
19	hsa-mir-335	dbDEMC, PhenomiR
20	hsa-mir-302a	dbDEMC, PhenomiR
21	hsa-mir-499a	Literature
22	hsa-mir-193a	PhenomiR
23	hsa-mir-138	dbDEMC, PhenomiR, miRCancer
24	hsa-mir-302c	dbDEMC, PhenomiR
25	hsa-mir-195	dbDEMC, PhenomiR
26	hsa-mir-629	dbDEMC, miRCancer
27	hsa-mir-27b	dbDEMC, PhenomiR
28	hsa-mir-302b	dbDEMC, PhenomiR
29	hsa-mir-127	dbDEMC, miRCancer
30	hsa-mir-30d	dbDEMC, PhenomiR
31	hsa-mir-135a	dbDEMC, PhenomiR
32	hsa-mir-373	dbDEMC, PhenomiR, miRCancer
33	hsa-mir-98	dbDEMC, PhenomiR
34	hsa-mir-137	dbDEMC, PhenomiR, miRCancer
35	hsa-mir-18b	dbDEMC
36	hsa-mir-152	dbDEMC, PhenomiR, miRCancer
37	hsa-mir-93	dbDEMC, PhenomiR
38	hsa-mir-497	dbDEMC, miRCancer
39	hsa-mir-206	dbDEMC, PhenomiR
40	hsa-mir-320a	dbDEMC, PhenomiR, miRCancer
41	hsa-mir-372	Literature

42	hsa-mir-494	dbDEMC, miRCancer
43	hsa-mir-92b	dbDEMC
44	hsa-mir-483	Literature
45	hsa-mir-185	dbDEMC, miRCancer
46	hsa-mir-342	dbDEMC, PhenomiR
47	hsa-mir-219	Literature
48	hsa-mir-302d	dbDEMC, PhenomiR
49	hsa-mir-149	dbDEMC
50	hsa-mir-129	dbDEMC, PhenomiR, TCGA
