# Supplementary figures and images for: Inferring the Disease-Associated miRNAs Based on Network Representation Learning and Convolutional Neural Networks
Source: Int J Mol Sci. 2019 Jul 25;20(15):3648. doi: 10.3390/ijms20153648 (PMC6696449; doi:10.3390/ijms20153648)

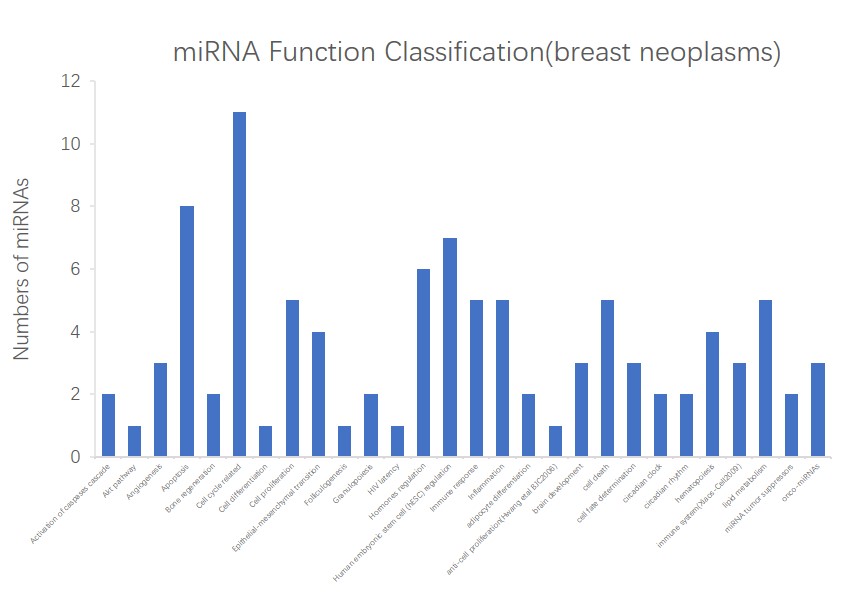

Supplement: Supplementary file 1 [file ijms-20-03648-s001.zip › breast neoplasms_miRNA functional enrichment analysis.jpg]

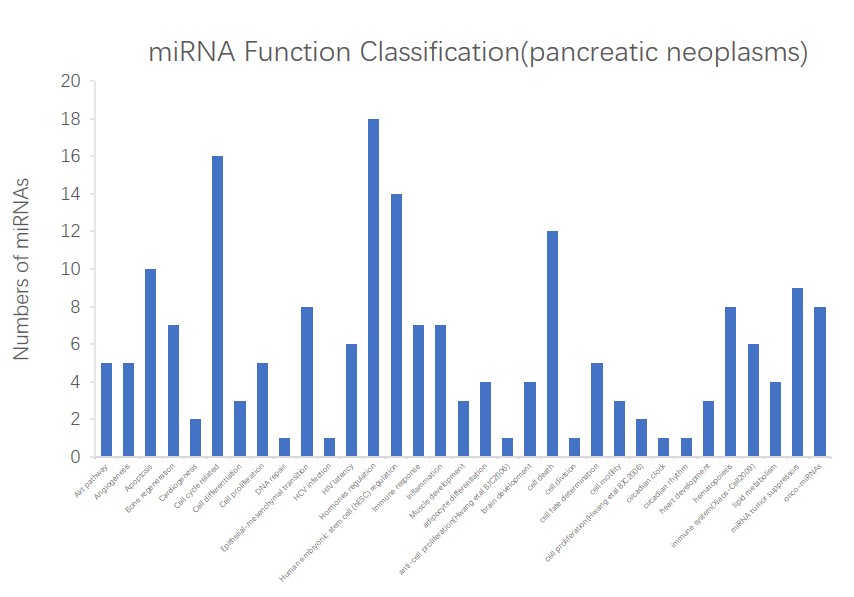

Supplement: Supplementary file 1 [file ijms-20-03648-s001.zip › pancreatic neoplasms_miRNA functional enrichment analysis.jpg]
